# Effects of Time-Restricted Feeding during Ramadan on Dietary Intake, Body Composition and Metabolic Outcomes

**DOI:** 10.3390/nu12082478

**Published:** 2020-08-17

**Authors:** Farhana Osman, Sumanto Haldar, Christiani Jeyakumar Henry

**Affiliations:** 1 Singapore Institute of Food and Biotechnology Innovations, 14 Medical Drive, Singapore 117599, Singapore; farhana_osman@sifbi.a-star.edu.sg (F.O.); sumanto_haldar@sifbi.a-star.edu.sg (S.H.); 2 Department of Biochemistry, National University of Singapore, 8 Medical Drive, Singapore 117596, Singapore

**Keywords:** Ramadan fasting, dietary intake, health outcomes, body composition, glucose homeostasis, lipid profile

## Abstract

Ramadan fasting is a form of time-restricted feeding which combines a fast and feast period daily for a duration of one month every year. During Ramadan, Muslims abstain from food and drink consumption from dawn till sunset and this change in the meal schedule and frequency results in significant changes to the composition of the diet, such as energy and nutrient intake. These changes in dietary habits and their corresponding effects on cardiometabolic disease risk are compiled in this review. Ramadan fasting shows limited benefits to body composition via reductions in body mass in both healthy and obese individuals, although the results are often found to be transient and heterogeneous. There is, however, a more consistent improvement in blood lipid profile during Ramadan fasting, which often lasts beyond the Ramadan period. The results for glucose homeostasis, on the contrary, are more conflicting and inconclusive. The heterogeneity in the findings from the various studies can be generally attributed to cultural variations in dietary habits, differences in the duration of fasting due to seasonal/climatic differences at various geographical locations, age, gender and socioeconomic status, as well as other health and lifestyle factors of the various study populations.

## 1. Introduction

Ramadan is the holiest month for Muslims all around the world, whereby the following of its practices is obligatory for all healthy Muslims. Fasting during the month of Ramadan is one of the five essential pillars of Islam, and this practice is considered an integral part of the faith. Globally, over one billion Muslims fast during the month of Ramadan [1], and the total time of fasting ranges from less than 12 h to as much as 19 h each day [2]. Fasting during the month of Ramadan originated in the seventh century to commemorate the month that Prophet Mohammed received divine revelation [3] and its purpose is to attain spiritual growth, better self-control and learn kindness [4].

Ramadan fasting is a form of “time-restricted feeding”, which combines fast and feast periods daily for a duration of one month every year. Ramadan fasting constitutes abstaining from food and drink consumption from dawn till sunset, which is typically followed by a feast. In addition to changes in the timing of food and fluid intake, Ramadan fasting results in significant changes to both energy balance and the composition of the diet [5]. The study of such changes to dietary habits and the effect of these alterations on human physiology is thus of importance.

Due to the unique nature of Ramadan fasting, although there has been extensive research done about it, the effects on human health are often contradictory, as Ramadan fasting is practised in different ways in different populations [5]. There are also differences in the physical activity levels of the various study populations and in the climate of the various countries, as well in the design of the various studies [5]. Therefore, it is important to investigate how the dietary habits of different populations during Ramadan impact various aspects of health.

In recent years, there has been a resurgence of interest in time-restricted feeding in relation to weight management, metabolic health and chronic disease risk [6,7,8,9]. The Ramadan fasting period offers a unique form of time-restricted feeding. Given the numerous metabolic and biological changes that occur during the Ramadan fasting period, it was deemed necessary to undertake a review to provide a source of information to those participating in the Ramadan fasting period. Inevitably, this meant the review focuses on the most relevant changes that occur during this period, notably changes in dietary habits, body composition and metabolic parameters.

In fact, there has been a lack of comprehensive reviews linking dietary changes to health outcomes during the Ramadan period. Therefore, the objective of the review was to compile various research done on these outcomes during Ramadan fasting. In particular, this paper aimed to focus on the effects of Ramadan fasting on the changes in fluid intake, dietary pattern, energy and macronutrient intakes, as well as changes in body composition, glucose homeostasis and lipid profile.

## 2. Materials and Methods

A systematic review of the published literature was conducted according to PRISMA (Preferred Reporting Items for Systematic reviews and Meta-Analyses) guidelines in two databases: PubMed and Google Scholar. The search terms used were “Ramadan fasting” AND “fluid intake”, “hydration status”, “dietary habits”, “nutrient intake”, “energy intake”, “body composition”, “body mass”, “weight”, “body fat percentage”, “obesity”, “glucose homeostasis” and “lipid profile”, respectively. The studies were first screened by title and abstract. Those that were relevant were then further screened by full text according to the inclusion and exclusion criteria.

Only peer-reviewed research published in scientific journals measuring the effect of Ramadan fasting on selected parameters, such as dietary habits, body composition and metabolic parameters, were included in this study. Publications with data from healthy individuals were included, as well as those investigating glucose homeostasis and fluid intake in individuals with type 2 diabetes or metabolic syndrome. Studies involving surgery, all other illnesses or illnesses measuring other parameters were excluded. Publications with obese individuals were analysed and discussed separately. Any available data on physical activity were extracted for analysis but studies involving only a sub-group of athletes were excluded.

**Figure 1:**
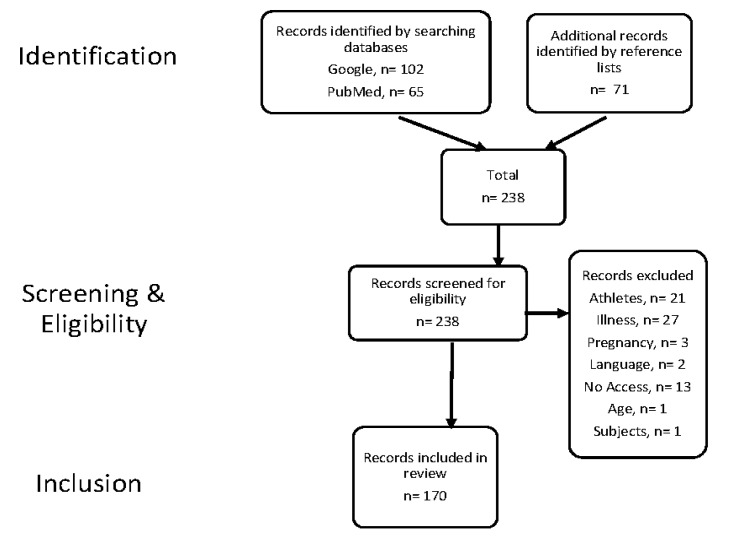
Flow diagram showing study selection based on inclusion and exclusion criteria.

Original research papers with studies containing fewer than nine volunteers were excluded from this systematic review, as well as those involving medications. We excluded studies involving volunteers who were children, pregnant or breastfeeding. Studies were also excluded when the full text of the publication had no English translation or if there was no full text available. The flow-diagram showing study selection process is shown in Figure 1. Meta-analyses or previously published systematic reviews were discussed under relevant sections to contextualise the findings from original papers.

## 3. Changes in Dietary Intake

### 3.1. Fluid Intake

It is widely believed that we need to consume approximately seven to ten glasses of water per day. Given that water intake is highly restricted within the period of Ramadan, in the first phase of this review, we will focus on the impact of fluid restriction on the levels of dehydration. Studies indicate that Ramadan fasting results in lower water intake during the month [10,11]. This potentially causes an alteration in kidney function, whereby water is reabsorbed by the body instead of being excreted in order to maintain fluid balance [11]. As such, there is effective water conservation during Ramadan fasting, by maximising urinary concentration as well as decreasing obligatory urine output [1,12]. However, prolonged dehydration induces a degree of stress on the concentrating ability of the kidneys and affects the efficiency of the water conserving mechanism [13]. The resulting hydration status of those who fast is still unclear, as there are conflicting findings regarding their level of dehydration.

Hydration status is commonly measured by serum osmolality and urine specific gravity [11]. In a study by Ibrahim et al. of 18 healthy young men during Ramadan fasting, it was found that serum osmolality levels were within the normal range, as the homeostatic mechanisms kept serum osmolality at normal levels [11]. Hosseini et al., Mustafa et al. and Dikme and Fasting also showed that there was no significant difference in serum osmolality before and during Ramadan fasting [12,14,15]. Trabelsi et al. investigated the effect of Ramadan fasting on body water levels and showed that there was no difference in total body water before and after Ramadan [16].

However, Meo and Hassan found haematocrit, plasma osmolarity and haemoglobin to be increased during Ramadan fasting and thereby possibly indicating dehydration [1]. This is in addition to an increase in urine osmolality reported in type 2 diabetics who fasted during Ramadan [17]. Furthermore, a comparison of urine specific gravity before and during Ramadan fasting by Ibrahim et al. showed that there indeed was dehydration during Ramadan fasting [11]. This state of dehydration is often caused by the fact that individuals have the same water intake habits before and during Ramadan fasting, thereby not drinking more water during non-fasting hours and eventually having lower fluid intake [11,18].

A study investigating the effect of Ramadan fasting on serum osmolarity in females showed that there was no significant change in osmolarity levels and total water when the subjects exercised regularly during Ramadan fasting, but there was a decrease in some electrolytes [14]. In a study by Ramadan et al., it was found that there was a significant increase in plasma osmolarity in those who were sedentary compared to active individuals after a month of Ramadan fasting [19].

These studies reveal that the hydration status of those who fast is dependent on their water intake habits and physical activity status. Serum osmolality levels are likely to remain in the normal range during the month of Ramadan, with increased water intake during non-fasting hours and regular exercise. Nonetheless, while Ramadan fasting has been shown to result in inadequate fluid intake in some studies, it is not clear whether it causes acute dehydration or chronic hypohydration due to the abovementioned factors that play a role in determining the hydration status of those who fast [13]. Moreover, negative water balance that may be caused by Ramadan fasting does not seem to have any detrimental health effects in the long term [12,13].

Finally, in terms of renal function parameters, studies investigating the effect of Ramadan fasting show that there was a significant reduction in serum creatinine, estimated glomerular filtration rate (eGFR) and urinary albumin/creatinine ratio in healthy individuals, but the decline was within the normal range [20]. In Muslim patients with type 2 diabetes, there was also a significant decrease in eGFR after Ramadan fasting [21]. In patients with chronic kidney disease (CKD), one study showed that there was an increase in serum creatinine in those who fasted [22], while no significant change was observed in another study [23]. Conversely, it was shown that serum creatinine significantly decreased and eGFR significantly increased in those who fasted with CKD [24]. Once again, the data show inconsistencies and the findings are generally dependent on the hydration levels during non-fasting hours, as well as other lifestyle factors during the fasting and non-fasting periods.

### 3.2. Food Intake Pattern

During Ramadan fasting, there is a variation in the frequency, timing and composition of the meals eaten [1]. However, a common pattern during fasting is one meal taken before dawn (“Suhoor”) and the fast is broken by a second meal at sunset (“Iftar”), which is typically a larger meal [25]. As the number of meals consumed is reduced from three or more to two meals daily, the overall amount and types of each meal eaten are changed. This in turn also affect energy and nutrient intake [25]. Due to the latitude and longitude variations between the various nations where the primary data were obtained, the duration of fasting between sunrise and sunset varies. It is therefore a difficult challenge to integrate food intake pattern and nutrient intake to generate comparable results. Some typical examples of pre-dawn “Suhoor” and post-dusk “Iftar” meals are shown in Table 1 [26,27] which reveals a diverse food intake pattern based on geographical location and culture.

The change in the composition of meals eaten during Ramadan is expected to contain more carbohydrate-rich foods in the form of fruits, juices and dates [1]. In a study of 366 Ghanaian adolescents, it was found that the consumption of milk and vitamin A-rich fruits increased during Ramadan, while there was a lower consumption of nuts, dark leafy vegetables and legumes [28]. On the other hand, in a study of 160 Iranian subjects, it was found that there was a higher consumption of fruits and vegetables during Ramadan, whereas the consumption of meat, dairy products and cereals decreased significantly [29]. Thus, there is a large variability between cultures and various geographical locations in the changes in dietary patterns that occur during Ramadan.

### 3.3. Macronutrient and Energy Intake

Ramadan fasting has a unique effect on dietary habits, whereby changes in the composition of the meals eaten directly affect nutrient and energy intake for a period of a month. With diabetes and cardiovascular diseases being widely prevalent in Muslim communities, it is important to elucidate the extent of the effect Ramadan fasting has on nutrient and energy intake and the metabolic consequences of such changes.

When comparing nutrient intake, some studies have found an increased intake of fat during Ramadan fasting [30,31,32,33,34,35,36,37]. In a cross-sectional study involving 173 Saudi families that reported weight gain during Ramadan, it was found that 40% of the subjects attributed their weight gain to the increased consumption of foods rich in fats and carbohydrates [27]. However, other studies suggest that there is a decrease in fat intake during Ramadan fasting [38,39]. These equivocal results on fat intake must interpreted with caution. As such, these differences may be of marginal longer-term metabolic relevance. Furthermore, Savas et al. found that there was no change in the eating behaviours of obese women during Ramadan fasting [40] whereas Khaled et al. showed that, although meal frequency was decreased, there was an increase in the consumption fat and dietary cholesterol by obese subjects during Ramadan fasting [41]. The effects of Ramadan fasting on nutrient intake are listed in Table 2.

The observed differences in nutrient intake suggest that meal compositions are different across countries and cultures. This was also noted by Karaagaoglu and Yucecan, who found that, while the pre-dawn meals consisted of food that was usually eaten at breakfast, the meals at the breaking of fast seemed to be much more variable [42].

Calorie intake is also affected by modifications in the timing and composition of food eaten, with a general trend in the first week of Ramadan towards a reduction in calorie intake, followed by a progressive increase through over consumption [1]. However, a study by Al-Hourani and Atoum on 57 female subjects found that the average energy and nutrient intakes before and during Ramadan were not significantly different [25], with this result being consistent with many other studies [30,31,43] and contrary to the idea that those who fast tend to consume more food than usual when breaking fast [25]. Indeed, some studies did show increases in total energy intake during Ramadan including in Saudi, Moroccan, Iranian and Turkish subjects [33,35,44,45,46,47,48], whereas other studies found a significant decrease in energy intake [32,38,49,50]. These contradictory results can again be attributed to the unique food habits of the different countries and cultures [25].

## 4. Changes in Body Composition

Since the food intake pattern is significantly altered, whereby food intake is restricted for about twelve hours, the fundamental question is whether this time-restricted feeding affects body composition and metabolic outcomes. In this subsequent section, we will discuss differences in body composition during Ramadan fasting compared with other times.

Studies investigating the effect of Ramadan fasting on body mass and body fat percentage show heterogeneous results, with some indicating decreases in body mass and body fat percentage [18,51,52,53,54,55,56,57], whereas others show no changes in body composition [31,58,59,60,61]. The heterogeneity of the results can be explained by a various demographic factors of the study participants. We have therefore listed the findings as per various sub-groups listed below.

### 4.1. Normal Weight

The effect of Ramadan fasting on body composition and body mass depends largely on the energy intake and expenditure of the subjects during the Ramadan period [5,62]. The findings from the various studies are listed in Table 3. In a study by Racinais et al., it was observed that 11 Qatari males of normal weight showed similar total daily energy expenditure before, during and after the month of Ramadan [60]. As a result, there was no significant change in body mass or composition reported. Similarly, Harder-Lauridsen et al. studied 10 men of normal weight in Denmark and found no change in body mass or composition before or after Ramadan [58]. The maintenance of body composition was found to be due to a balance in total energy intake and energy expenditure by the study participants [58].

Other studies have found a reduction in body mass and fat percentage and these discrepancies can be attributed to varying practices with regard to energy intake and expenditure during Ramadan fasting by different populations [62]. Just as some studies have shown a decrease in body weight and fat percentage during Ramadan [25], it has also been found that the effect stayed till the end of Ramadan [63]. Shehab et al., investigating the effects of Ramadan fasting on body weight, found that there were losses both in body weight and waist circumference immediately following Ramadan [64], although all the above mentioned studies had limited follow-up periods beyond Ramadan. A meta-analysis of eighty-five studies showed that Ramadan fasting can cause a significant small reduction in weight in healthy adults that has direct associations with fasting duration and correlates with season and country [65].

However, a number of studies showed that, by four to five weeks after Ramadan, the decrease in body weight and fat percentage had returned to pre-Ramadan levels [55,66,67]. Sadeghirad et al. did a systematic review and meta-analysis of thirty-five studies determining the changes in body mass because of Ramadan fasting and showed that weight loss did not last for more than two weeks after Ramadan [68]. It was shown that most of the subjects who lost weight regained it within two weeks and only a small amount of weight loss was maintained after Ramadan compared to the start of it [68].

This may be attributable to the fact that Ramadan fasting lasts for a period of only one month and thus there is limited impact on body mass and fat percentage. As can be seen from Table 3, studies showed less than a 5% reduction in body mass and fat percentage due to Ramadan fasting. Though the observed effect is small, it is indicative of a possible positive impact over a longer period of time. Therefore, it is worthwhile to investigate the cumulative effect of Ramadan fasting on body composition over a number of years to ascertain its clinical impact.

Studies involving physically active individuals during Ramadan fasting is of importance to understand the link between energy expenditure and body composition. Most studies in physically active individuals who exercised during Ramadan fasting showed a decrease in body mass [19,72,73,74]. This is likely due to dehydration and a decrease in caloric intake while maintaining training [72].

A similar trend of decreases in body fat percentage was noticed in most studies investigating the effect of Ramadan fasting on body composition in physically active individuals [16,72,73]. The decrease in body fat percentage is likely due to the increased utilisation of stored body fat as an energy substrate [72]. In the studies where an absence of weight change and body fat percentage was shown, the discrepancy can be explained by differences in dietary intakes, exercise regimens and methods of body fat measurements [72].

It can be observed form the results that there is a more consistent link between Ramadan fasting and an improvement in body composition in physically active individuals. This shows that physical activity is an important part of energy expenditure during Ramadan fasting and suggests that the greatest benefit from the fasting regime can only be obtained when volunteers undertake Ramadan fasting in combination with physical activity. Overall, it has been found that Ramadan fasting results in variable amounts of weight loss and changes in body composition in normal weight individuals, but the changes are often transient.

Other factors could also play a role in determining the effect of Ramadan fasting on body composition and mass, including gender and age [1,52]. Norouzy et al. assessed the effect of Ramadan fasting on body composition and found that males and younger individuals had a greater reduction in body weight as well as body fat percentage [52]. López-Bueno et al. studied the influence of age on body composition during Ramadan fasting and showed that there was a more pronounced reduction in body fat percentage of women over thirty years of age [51]. Husain et al. studied tropical Asiatic Muslims and found that females lost more body weight and subcutaneous fat than males [49].

Yucel et al. studied the effect of Ramadan fasting on abdominal fat distribution and found that there was a significant reduction in the visceral fat area within the sub-groups of females and individuals in their twenties [75]. Yeoh et al. showed that there was a reduction in visceral adiposity only amongst females when the results were stratified by gender [34]. In contrast, Syam et al. found that protein body mass did not significantly change due to Ramadan fasting and that weight loss was not variable according to gender [55]. It is likely that younger individuals have greater reductions in body fat percentage, as it is a factor affected directly by personal activity [75]. Young people are generally more physically active and have higher basal metabolism [75]. Additionally, many of the studies showed that females experienced a greater change in body composition, as they are likely to be more physically active than men during Ramadan fasting [75].

### 4.2. Overweight and Obese

Ramadan fasting is often practiced as a lifestyle modifier in tackling obesity among Muslim communities. The decreased frequency of meals is expected to result in lower caloric intake and eventual weight loss at the end of Ramadan. However, the effect of Ramadan fasting on the body weight and composition of obese individuals is heterogeneous, with some studies showing a loss in body weight only [41,76,77,78,79], whereas other studies show a change in body fat percentage as well [53,80,81,82,83]. This heterogeneity in the findings could be due to different dietary habits between different cultures during the month of Ramadan. The various findings are listed in Table 4.

In a study where a medium calorie balanced diet was prescribed to 28 overweight males during the month of Ramadan, fasting resulted in a significant decrease in body weight [84], indicating that the composition of the diet is important. Furthermore, a meta-analysis of 70 publications by Fernando et al. showed that there was a significant positive correlation between starting BMI and loss of weight during Ramadan fasting [66]. In the same report, there was also a significant decrease in body fat percentage among obese individuals after Ramadan as compared to before Ramadan (−1.46 (95% confidence interval: −2.57 to −0.35) %, *p* = 0.010) [66]. Therefore, Ramadan fasting, when coupled with a balanced diet, can result in a positive change in obese individuals. However, the reductions in weight and fat mass were transient and body fat percentage returned to pre-Ramadan levels 2 to 5 weeks after the end of Ramadan. The authors explained that one possible reason for the greater weight loss in people with a higher BMI is that they have bigger glycogen stores than individuals with a lower BMI, and thus are likely to lose more body water during Ramadan fasting. Furthermore, obese individuals lose less weight as fat-free mass as compared to individuals of normal weight, thereby resulting in lower body fat percentage in the obese [66].

Therefore, Ramadan fasting results in weight loss amongst obese individuals with a possible effect on body fat percentage as well. However, most studies show that the changes in weight and body fat percentage in overweight and obese individuals are often transient and return to pre-Ramadan levels within a few weeks after the end of the Ramadan period.

## 5. Metabolic Parameters

In many regions of the world, notably Asia, the Middle East and parts of Africa, many residents are highly susceptible to pre-diabetes, diabetes and cardiovascular diseases. Therefore, for the subsequent parts of this review, we will discuss how Ramadan fasting influences glucose and lipid metabolism before, during and after Ramadan in studies from various geographical locations and with distinct dietary and lifestyle habits. We will present data not only from healthy individuals but also those at risk of various chronic conditions (e.g., obesity, type 2 diabetics, etc.).

### 5.1. Glucose Homeostasis

Some studies show that there is an increase in fasting blood glucose levels during the Ramadan fasting period [36,86], while other studies show decreases or no change [35,50,59,86,87,88,89,90,91,92,93,94,95,96]. These findings are listed in Table 5. The increased fasting blood glucose levels in Thai women, as shown by Ongsara et al., in addition to their dietary change, may be due to the decreased frequency of exercise during and after Ramadan compared with before Ramadan [86]. This is supported by research showing that exercise increases insulin sensitivity and glucose uptake into peripheral tissues [86,97]. A shift in the intake of carbohydrates from complex to more simple sugars could have also increased the levels of fasting blood glucose [36].

Furthermore, the measurement of fasting blood glucose at different times of the day could have resulted in the observed higher fasting blood glucose levels during Ramadan. In most of the studies, before the start of the Ramadan period, fasting blood glucose levels were measured in the morning, whereas during Ramadan, fasting glucose levels were measured in the evening after the daytime fast [50,87,90]. This difference in the time when fasting blood glucose was measured is of significance, as the glucose values in the evening may be higher due to diurnal variation [98]. Thus, fasting blood glucose measured after an overnight fast is not necessarily equivalent to that taken in the evening after Ramadan fasting and their comparison is inaccurate. Furthermore, in addition to the diurnal variation in glucose metabolism, the durations of fasting periods were also different.

In a study of twenty healthy males during Ramadan fasting, it was revealed that there were significant decreases in fasting plasma insulin and glucose by 52.8% and 12.3, respectively (*p* < 0.01) [100] and, consequently, a decrease in insulin resistance using HOMA-IR (Homeostatic Model Assessment of Insulin Resistance, *p* < 0.01), which the authors believed was due to calorie restrictions. However, this study also showed reductions in fasting adiponectin by 45.6%, which was in fact positively associated with decreases in body weight. These results suggested that Ramadan fasting can help with maintaining glucose homeostasis among young people [100].

In type 2 diabetics, however, Ramadan fasting leads to much more variable changes, with some studies finding negligible changes in glycaemic control [83,101,102,103,104], whereas others show improvements [88,105,106,107]. These findings are listed in Table 6. The effects of no change in glycaemic control have often been substantiated with evidence showing that HbA1c values and fructosamine did not change during Ramadan fasting [103,108,109]. However, the time frame for the HbA1c analyses of about 4 weeks during the month of Ramadan is not long enough to give an appropriate reflection of glycaemic control, thus leading to uncertainties in the findings.

On the other hand, continuous glucose monitoring (CGM) may be a better reflection of glycaemic control within the time frame of Ramadan. Pallayova et al. studied glucose homeostasis before, during and after Ramadan with CGM in non-diabetic adults and reported a higher hyperglycaemic area under the curve after Ramadan than before and during [111]. Hassanein et al. investigated the risk of Ramadan fasting in diabetics through the use of CGM and found that there was a significantly higher occurrence and prolonged duration of hypoglycaemia during Ramadan fasting [112]. Similarly, there are other studies reporting acute adverse events, such as severe hyperglycaemia or hypoglycaemia, in diabetic patients during Ramadan [113,114]. A population-based study of diabetes and its characteristics during Ramadan fasting in 13 countries revealed that severe hypoglycaemia was significantly more common during Ramadan than in other months (type 1 diabetes, 0.14 vs. 0.03 episodes/month, *p* = 0.0174; type 2 diabetes, 0.03 vs. 0.004 episodes/month, *p* < 0.0001) [113].

These results suggest that Ramadan fasting is likely to cause substantial changes in glycaemic control in type 2 diabetics and therefore their meal patterns require particular attention. This is because when food is eaten at the breaking of fast, blood glucose increases drastically, followed by overnight hyperglycaemia due to late-night eating, while the meal taken before dawn causes prolonged glucose decay in the daytime [115]. This pattern of glycaemic increase and decrease is more common among diabetic patients under medication and in patients who have poor control of their condition or do not comply with lifestyle recommendations [115,116]. Hence, from a religious and cultural point of view, Ramadan fasting is encouraged in healthy individuals and those with health conditions are exempted from this fasting ritual.

It should be noted, however, that previous studies of Ramadan fasting in type 2 diabetics were often influenced by recall bias, and up to two-thirds of the patients either maintained or increased their insulin dosing during Ramadan, which could be one of the reasons for the increased incidence of severe hypoglycaemia [105,117]. There are, however, some studies which indicated that the prevalence of severe hyperglycaemia or hypoglycaemia during Ramadan was low among diabetic patients [36,105,118]. A study in Pakistan investigating the glycaemic levels of patients with diabetes during Ramadan fasting showed that the incidence of hyperglycaemia was 19.8% and hypoglycaemia was 21.7% and that less than 10% had major hypoglycaemic and hyperglycaemic episodes [119].

Therefore, the results suggest that Ramadan fasting has variable impacts on glucose homeostasis in both healthy and diabetic individuals and that the specific food choices and lifestyle factors, as part of the fasting regime, could also play important roles. In order to achieve better glucose homeostasis, it is recommended that there be an increased intake of fruits and vegetables high in complex carbohydrates and a decreased intake of simple sugars [86].

### 5.2. Lipid Profile

Studies investigating the effect of Ramadan fasting on the lipid profile have been promising, as most of them show improvements in high density lipoprotein (HDL) and low density lipoprotein (LDL) cholesterol levels after one month of Ramadan [30,44,50,64,120,121,122,123,124,125,126,127,128,129,130,131,132]. The various studies are listed in Table 7.

The increase in HDL cholesterol levels can be explained by the post-prandial lipaemia changes that occur during Ramadan fasting [126]. Since the amount of post-prandial lipaemia has been shown to be of great importance to lipid metabolism and HDL levels in plasma [146], the larger magnitude and duration of lipaemia during the gorging meal, followed by longer absorptive-free hours during fasting, results in a net balance that is more efficient in the metabolism of triglyceride (TG) rich lipoproteins [126]. This could be the reason for the observed increased plasma HDL cholesterol levels in many of the studies.

In addition, the effect of Ramadan fasting on lipid profile was shown to extend to even after Ramadan. Shehab et al. found that there was a significant increase in HDL and a decrease in LDL cholesterol levels a month after Ramadan fasting [64]. These results suggest that Ramadan fasting may be a suitable lifestyle modification to improve blood lipid profile [64,129,147]. Thus, future studies evaluating the effect of Ramadan fasting in people with lipid disorders is of importance [129].

However, a cohort study by Ziaee et al. of 81 students found that there was a decrease in HDL and an increase in LDL levels during Ramadan fasting [90]. The contradiction in the results may be due to a change in the dietary regimen during Ramadan, decreased physical activity and cultural influence on dietary patterns [90,133,148]. Haghdoost et al. studied the connection between physical activity and Ramadan fasting on the lipid profile and found that total cholesterol levels decreased significantly during and after Ramadan for subjects who were physically active while fasting (by −12.24 and −8.4mg/dL, respectively) [74]. However, the patterns of changes in HDL and LDL cholesterol were more or less comparable in both physically active and inactive groups [74]. This finding shows that the observed reduction in total cholesterol was probably due to reductions in TG levels and physical activity may have been the effect modifier.

In fact, several studies showed a decrease in TG levels during Ramadan fasting [44,64,74,79,121,124,133,134,135,136], although other studies found no change [90,122,123,125,129,131,137]. Two studies did observe an increase in serum TG, which may be due to the consumption of foods rich in carbohydrates accompanied by less physical activity during Ramadan [90,129]. This is linked back with studies showing that people are more likely to consume carbohydrate-rich foods during Ramadan [33,90,149,150,151]. Hallak and Namoni showed a positive correlation between serum triglyceride levels and sugar intake during Ramadan [135,152]. The increase in blood triglycerides with high sugar consumption was also observed by Albrink and Ullrich [135,153]. Alternatively, the lipolytic effect of fasting for long periods of time may result in higher serum triglyceride levels at the end of Ramadan fasting [131,154].

In cases where a decrease in TG levels was found, it could be due to the increased consumption of monounsaturated and polyunsaturated fatty acids, as well as the decreased consumption of saturated fatty acids [64]. This was likely to be so in a study from Morocco, where a significant decrease in triglyceride levels was observed even after the month of Ramadan [44,64]. It seems as though the type of fat intake during Ramadan determines the resulting blood cholesterol level [131].

Thus, the variations in the lipid profile before and after Ramadan in healthy individuals are often attributed to differences in dietary habits between countries, like that of the classic Mediterranean diet in Morocco and the diet high in saturated fat in affluent countries of the Arabian Gulf states [64,137]. These differences could also be explained by the fact that the duration of fasting differs among countries depending on the seasonal climate following the lunar calendar [137]. This reiterates the fact that Ramadan fasting and the breaking of fast is practised differently in different populations and has varying impacts on the health outcomes of those who fast.

Overall, there is a potential for a favourable impact on cardiometabolic risk factors due to improved lipid profile during Ramadan fasting. A meta-analysis by Kul et al. found that Ramadan fasting resulted in lower LDL cholesterol levels in both males and females, as well as all of the subjects [155]. Additionally, HDL cholesterol levels were also increased among females, whereas triglycerides (TGs) decreased to a small extent in among males only. Overall, this caused a rise in total cholesterol [155]. Similarly, a review by Mazidi et al. found that the majority of studies reported an increase in HDL levels, a decrease in triglyceride levels and no change or a decline in total cholesterol and LDL levels [156].

Beyond blood lipid profile, a systematic review investigating the effect of Ramadan fasting on cardiometabolic risk factors showed that, generally, the effects may be described as beneficial or neutral, although there were some exceptions [157]. In fact, three studies investigating the effects of Ramadan fasting on blood pressure, a risk factor for cardiometabolic diseases, reported a significant drop in systolic blood pressure during Ramadan fasting, while most studies revealed no change in blood pressure [157]. Furthermore, a prospective observational study showed significant changes in some cardiovascular risk factors during Ramadan fasting, including an improvement in 10-year coronary heart disease risk (based on Framingham risk score) and a reduction in systolic blood pressure, body mass index and waist circumference, although C-reactive protein (CRP) and homocysteine were not significantly different [130]. Ramadan fasting has also been shown to have some beneficial effects on endothelial function, like improved nitric oxide availability, which can affect cardiometabolic risk [158]. Some studies investigated the effect of Ramadan fasting on inflammatory markers and showed that there was a significant reduction in CRP levels, the attenuation of proinflammatory cytokines, a decrease in IGF-1 and IL-2 levels, an increase in adiponectin, a decrease in TNF-α and a reduction of serum amyloid A and protein carbonyl group levels [159,160,161,162,163]. However, the effects are not conclusive and an improvement in cardiometabolic risk factors cannot be substantiated by current studies due to several limitations. This is because prospective studies carried out over a long period of time are lacking and the studies are generally small in size [157].

#### Overweight and Obese

Given that overweight and obese individuals are likely to respond differently compared to normal weight individuals to various dietary interventions, in this section we will specifically discuss the changes in lipid profile due to Ramadan fasting in overweight and obese individuals. A study conducted on 103 obese individuals in Egypt showed that Ramadan fasting resulted in a significant improvement in total cholesterol, total triglycerides, high density lipoprotein, low density lipoprotein, TC/HDL ratio, LDL/HDL ratio, lipoprotein a, apolipoprotein, apolipoprotein B levels and coagulation parameters, which continued for four weeks after fasting [164]. Similarly, in another study on 60 obese women with type 2 diabetes in Algeria, Ramadan fasting resulted in a significant improvement in glucose homeostasis, although total cholesterol, triglycerides and LDL levels also increased significantly [107]. Ramadan fasting has also been shown to increase the genetic expression of anti-inflammatory, antioxidant and metabolic regulatory genes in overweight and obese individuals [79,165].

Leptin levels are important in regulating the long-term deposition of fat but the effect of Ramadan fasting on leptin levels is not well established [166]. Kassab et al. showed that serum leptin levels increased significantly by 37% during Ramadan fasting in obese individuals [166]. In contrast, Alzoghaibi et al. observed a significant drop in plasma leptin levels during Ramadan fasting [167]. Even though the loss of weight typically results in a drop in serum leptin levels, in the case of obese individuals, increased fat oxidation and increased serum leptin were correlated [168]. As such, it is not clear how Ramadan fasting affects serum leptin levels and the eventual deposition of fat in obese people and more future studies in this area are recommended.

## 6. Discussion

Ramadan fasting is a mandatory practice for all healthy Muslims and it is one of the five pillars of Islam. Its uniqueness as a type of time-restricted feeding results in dietary habits that are unlike any other type of fasting or time-restricted feeding diet. In particular, Ramadan fasting leads to changes in nutrient intake and the composition of the diet, although the changes are variable based on location, as well as culture.

The effect of Ramadan fasting on health outcomes is an important investigation to understand the link between abstaining from food and water during daylight hours for an entire month and human physiology. Furthermore, the high occurrence of cardiovascular diseases in Muslim communities makes these investigations particularly pertinent [157]. Whilst recognising that fasting during Ramadan is not an experimentally rigorous time-restricted feeding regimen, its advantage is the availability of a very large number of datasets. Recognising potential bias, it is fair to assume that, due to the religious conviction, most will comply with time-restricted fasting.

Ramadan fasting has been shown to be of some benefit, as it causes significant reductions in body mass in healthy and obese individuals and, in some cases, reductions in body fat percentage as well. This can be explained by the fact that the human body adapts during fasting to slow down basal metabolism and utilise fats more efficiently in conditions of negative energy balance [131]. The most promising health outcome due to Ramadan fasting appears to be an improvement in lipid profile, which often extends even after the month of Ramadan fasting. An improvement in lipid profile could be beneficial for the cardiovascular system, as HDL is protective against coronary heart disease [95,169].

The results for glucose homeostasis are more conflicting and there are only a few studies showing an improvement in fasting blood glucose levels and, given that fasting measurements are often measured at different times pre- and post-Ramadan fasting, this is of some concern in the interpretation of these findings. Furthermore, inconsistencies in the results of the various studies investigating the effects of Ramadan fasting on health outcomes can be explained by the abovementioned limitations, such as differences in the duration of fasting caused by seasons and climate, variations in dietary habits and socioeconomic status, different study methodology and the age and gender of the individuals, as well as other health and lifestyle factors [157].

When comparing Ramadan fasting to other types of intermittent fasting, such as alternate day fasting, time-restricted feeding (e.g., feeding during daylight hours only) and modified fasting regimens, it can be observed that the effect on body mass and composition is more pronounced in the other types of intermittent fasting [170]. Many of the studies involving other types of intermittent fasting show weight loss that is less transient than Ramadan fasting [170]. However, the effects of the other types of intermittent fasting on metabolic parameters are mixed and no more conclusive than Ramadan fasting [170].

One of the limitations of investigating the effect of Ramadan fasting on health outcomes is the lack of longer-term prospective studies. For example, the cumulative longer-term effects of Ramadan fasting for 1 month every year for several years are yet to be established. Indeed, this is an area of focus that should be investigated in future studies and is of significance in understanding the longer-term health implications of Ramadan fasting over a number of years. Another limitation of studies involving Ramadan fasting is that many of them do not have controls, thus resulting in further limitations to the data [157]. This is because it is problematic to compare fasting and non-fasting individuals or include randomisation in the study procedure [157].

Overall, this review shows that there are some aspects of Ramadan fasting that result in an improvement in health outcomes. The type of diet, in particular, is an important arbitrator of the effect of Ramadan fasting on health. In cases where there were positive changes in diet during Ramadan, there were observed improvements in health outcomes. In particular, it is recommended in Islam to have a balanced diet rich in fruits and vegetables. Studies that showed improvements in body composition or metabolic parameters were found to have fruit- and vegetable-rich diets, thus following these recommendations more closely [64,137]. On the other hand, studies of Ramadan fasting in countries with diets rich in saturated fat did not show favourable results [64,137].

Furthermore, it is recommended in Islam to have moderate food intake and be physically active during Ramadan. The positive effect of physical activity during Ramadan fasting can be observed in studies involving physically active individuals, where a decrease in body fat percentage was observed in most cases [16,72,73]. As such, there are many different conditions that play a part in Ramadan fasting and have varied effects on health outcomes. All these factors need to be kept in mind while following an optimal Ramadan fasting regime to obtain the maximal health benefits that this religious practice is meant to impart.

## Figures and Tables

**Table 1:** Food intake pattern during Ramadan in different countries around the world [26,27].

	Pre-Dawn Meal (Suhoor)	Breaking Fast Meal (Iftar)			
		Starters	Main	Others	Drinks
Algeria	- Couscous mixed with sultanas and buttermilk	- Chorba soup: tomatoes, vegetables, vermicelli and lamb neck - Borek: fried filo pastries with spinach and cheese/lamb and potato	- Salads - Stews - Bread	- Laham lalou: dried fruits cooked with fresh apple/pear and spices	
Bangladesh	- Chapatti - Lentil soup	- Seasonal fruits: watermelon, mango, guava, jackfruit	- Rice - Meat/fish - Lentils - Potato cakes - Kichiri: rice, lentils, onions, garlic, ginger		- Lassi
Egypt	- Ful madammas: warm dip made with broad beans - Eggs - Cheese - Bread	- Dates - Green salad - Dips	- Sambusak: pastries with cheese or meat filling - Mahshi: stuffed vegetables - Moloukhia: green leafy vegetable - Kebabs - Rice		- Milk
Saudi Arabia	- Rice - Salad - Cooked vegetables - Meat	- Dates - Meat soup - Sambosa: pastry filled with meat or cheese - Pastries	- Salad - Bread - Meat - Beans	- Sweets	- Coffee
Singapore	- Bread/Cereal	- Dates - Curry puff: fried pastries filled with potato/sardine - Traditional sweet snacks	- Rice - Curry - Vegetables - Rice porridge made with rice, meat, vegetables and spices.	- Fruits	- Bandung: sweet drink

**Table 2:** Effect of Ramadan fasting on energy, carbohydrate, protein and fat intakes.

Authors	Date	Country	Subjects (n)	Effect of Ramadan on Nutrient Intake
Rahman et al. [30]	2004	Bangladesh	20 males	No change in energy, carbohydrate or protein
				Increase in fat
El Ati et al. [31]	1995	Tunisia	16 females	Increase in protein and fat
Frost and Pirani [33]	1987	Saudi Arabia	15	Increase in carbohydrate, protein and fat
Yeoh et al. [34]	2015	Singapore	29	No change in energy, carbohydrate or protein
				Increase in fat
Lamine et al. [35]	2006	Tunisia	30	Increase in fat
Sadiya et al. [36]	2011	UAE	19	Decrease in protein, increase in fat
Bouhlel et al. [37]	2006	Tunisia	9	Decrease in carbohydrate and protein
				Increase in fat
Poh et al. [38]	1996	Malaysia	117	No change in protein. Decrease in carbohydrate and fat
Suriani et al. [39]	2015	Malaysia	84	Decrease in carbohydrate
Khattak et al. [43]	2013	Malaysia	30	No change in energy or macronutrient intake

**Table 3:** Effect of Ramadan fasting on body mass and fat percentage of normal weight individuals.

	Date	Country	Subjects (n)	Effect on Body Mass	Effect on Body Fat Percentage
Bouhlel et al. [18]	2008	Tunisia	9 males	Significant reduction of 1.8 kg	Significant reduction of 1.3%
López-Bueno et al. [51]	2015	Spain	62 females	Significant reduction of 1.6%	Significant reduction of 2.2%
Nachvak et al. [57]	2019	Iran	160 males	Significant reduction of 1.93 kg	Significant reduction of 0.3%
Norouzy et al. [52]	2013	Iran	240	Significant reduction of 2.2% in males and 1.4% in females aged ≤ 35 years	Significant reduction of 2.5% in males ≤ 35 years and 1.1% in males 36 to 70 years
Shruthi et al. [54]	2013	India	50	Significant reduction of 0.59 kg	Significant reduction of 3.155%
Syam et al. [55]	2016	Indonesia	43	Significant reduction of 0.874 kg	Significant reduction of 0.484 kg
Kocaaga et al. [69]	2019	Turkey	33 males	Significant reduction of 0.84 kg	Significant reduction of 1.32%
Amiri et al. [70]	2016	Iran	51	Significant reduction of 0.78 kg	Significant reduction of 0.49 kg
El Ati et al. [31]	1995	Tunisia	16 females	No significant change	No significant change
Harder-Lauridsen et al. [58]	2017	Denmark	10 males	No significant change	No significant change
Ramadan [59]	2002	Kuwait	16 males	No significant change	No significant change
Racinais et al. [60]	2012	Qatar	11 males	No significant change	No significant change
Finch et al. [61]	1998	UK	41	No significant change	No significant change
Al-barha et al. [71]	2019	Saudi Arabia	44	No significant change	No significant change

**Table 4:** Effect of Ramadan fasting on body mass, body fat percentage and visceral adiposity of overweight and obese individuals.

Authors	Date	Country	Subjects (*n*)	Effect on Body Mass	Effect on Body Fat Percentage	Effect on Visceral Adiposity
Khattak et al. [76]	2012	Malaysia	25	Significant reduction of 15.8 kg in males and 15.4 kg in females	No change	-
Khan et al. [77]	2002	Pakistan	10 males	Significant reduction of 3.2 ± 1.7 kg	-	-
Rohin et al. [78]	2013	Malaysia	27	Significant reduction of 0.95 kg for overweight subjects and 2.07 kg in obese subjects	No change	-
Celik et al. [80]	2014	Turkey	42	Significant reduction of 1.6 kg	-	-
Suriani et al. [81]	2015	Malaysia	48	Significant reduction of 1.76 kg	Significant reduction of 0.32%	Significant reduction of 0.75 kg
Khaled et al. [41]	2009	Algeria	276	Significant reduction of 3.12 kg	-	-
Madkour et al. [82]	2019	UAE	56	Significant reduction of 1.15 kg	Significant reduction of 1.22 kg	Significant reduction of 5.82 cm²
Ünalacak et al. [79]	2011	Turkey	10 males	Significant reduction of 2.9 kg	-	-
Radhakishun et al. [83]	2014	Netherlands	25	No change	Significant reduction of 2.5%	-
Salehi et al. [84]	2007	Iran	28 males	Significant reduction of 6%	-	-
Sezen et al. [53]	2016	Turkey	70	-	Significant reduction of 0.9 kg	Significant reduction of 0.5%
Ganjali et al. [85]	2016	Iran	24	Significant reduction of 2.2 ± 1.81 kg	-	-

**Table 5:** Effect of Ramadan fasting on fasting blood glucose levels in healthy subjects.

Authors	Date	Country	Subjects (*n*)	Effect of Ramadan Fasting on Fasting Blood Glucose Levels
Ongsara et al. [86]	2017	Thailand	65	Increase
Fakhrzadeh et al. [50]	2003	Iran	91	Decrease
Larijani et al. [87]	2003	Iran	115	Decrease
Sarraf-Zadegan et al. [89]	2000	Iran	50	No change
Ziaee et al. [90]	2006	Iran	81	Decrease
Azizi and Rasouli [91]	1987	Iran	9	No change
Kiyani et al. [92]	2017	Pakistan	80	Decrease
Ramadan [59]	2002	Kuwait	16	No change
Lamine et al. [35]	2006	Tunisia	30	No change
Beltaifa et al. [93]	2002	Tunisia	20	No change
Roy et al. [96]	2017	India	37 males	Decrease
Darzabi et al. [99]	2019	Iran	15 males	Decrease

**Table 6:** Effect of Ramadan fasting on fasting blood glucose levels in subjects with health conditions.

Authors	Date	Country	Subjects (n)	Health Condition	Effect of Ramadan Fasting on Fasting Blood Glucose Levels
Sadiya et al. [36]	2011	UAE	19	Metabolic syndrome	Increase
Shariatpanahi et al. [88]	2008	Iran	55	Metabolic syndrome	Decrease
Radhakishun et al. [83]	2014	Netherlands	25	Obesity	No change
Mafauzy et al. [101]	1990	Malaysia	22	Type 2 diabetes	No change
Lessan et al. [102]	2015	UAE	56	Type 2 diabetes	No change
Al-Hader et al. [103]	1994	Jordan	23	Type 2 diabetes	No change
Yarahmadi et al. [104]	2003	Iran	57	Type 2 diabetes	No change
Khatib and Shafagoj [106]	2004	Jordan	44	Type 2 diabetes	Decrease
Khaled et al. [107]	2006	Algeria	60	Obesity and Type 2 diabetes	Decrease
Norouzy et al. [110]	2012	Iran	89	Type 2 diabetes	Increase

**Table 7:** Effect of Ramadan fasting on lipid profile in healthy individuals.

Authors	Date	Subjects (n)	Effect of Ramadan on Parameters
Adlouni et al. [44]	1997	32 males	Increase in HDL
			Reductions in TC, TG and LDL
Akaberi et al. [129]	2014	43	Increases in TC, HDL and LDL
			No change in TG
			Reductions in LDL/HDL and TG/HDL
Akanji et al. [125]	2000	64	Increases in apo A-1, apo A-1/apo B, apo A-1/HDL
			No change in TC, TG or LDL
Al Hourani et al. [133]	2009	57 females	No change in TC, HDL or LDL
			Reduction in TG
Asgary et al. [134]	2000	50 males	Reductions in TC and TG
Dowood [131]	2004	60	No change in TC, TG, HDL or VLDL
			Reduction in LDL
Fakhrzadeh et al. [50]	2003	91	Increase in HDL
			Reductions in TC, TG and LDL
Furuncuoglu et al. [135]	2007	39	No change in HDL
			Reductions in TC and TG
Haghdoost and Poorranjbar [74]	2009	93	Reduction in TG
Haouari-Oukerro et al. [121]	2013	38 males	Increase in HDL
			No change in TC
			Reductions in TG and LDL
Hosseini and Hejazi [132]	2013	26 males	Increase in HDL
			Reductions in TC, LDL, VLDL, LDL/HDL and TC/HDL
Lamri-Senhadji et al. [120]	2009	46	Increase in HDL
			Reduction in LDL
Mahoob et al. [136]	1999	35 males	Reductions in TG, LDL, TC/HDL and LDL/HDL
M Maislos et al. [126]	1998	22	Increase in HDL
			No change in TC, TG, LDL or VLDL
M Maislos et al. [123]	1993	24	Increases in HDL, apo A-1
			No change in TC, TG, LDL or VLDL
			Reductions in TC/HDL and LDL/HDL
Mansi [122]	2007	70	Increase in HDL
			No change in TC or TG
			Reduction in LDL
Nematy et al. [130]	2012	82	Increase in HDL
			Reductions in TC, TG, LDL and VLDL
Qujeq et al. [128]	2002	83	Increase in HDL
			Reduction in LDL
Rahman et al. [30]	2004	20 males	Increase in HDL
			No change in TC, TG or LDL
			Reductions in TC/LDL and LDL/HDL
Saleh et al. [137]	2005	60	No change in TG, HDL or VLDL
			Reductions in TC and LDL
Shehab et al. [64]	2012	65	Increase in HDL
			No change in TC
			Reductions in TG and LDL
Temizhan et al. [127]	2000	52	No change in HDL
			Reductions in TC, TG, LDL and VLDL
Ünalacak et al. [79]	2011	20 males	No change in TC, HDL or LDL
			Reduction in TG
Zare et al. [124]	2011	32 males	Increases in HDL and HSP70
			Reductions in TC, TG, LDL, LDL/HDL and TC/HDL
Ziaee et al. [90]	2006	81	Increase in LDL
			No change in TC, TG or VLDL
			Reduction in HDL
Thannoun et al. [138]	2010	31	Increase in HDL
			Reduction in TC, LDL and TG
Marbut et al. [139]	2006	30 males	Increase in HDL
			Reduction in LDL
Akhtaruzzaman et al. [140]	2014	28 females	Increase in HDL
			Reduction in TC and LDL
Ismail et al. [141]	2014	31	Increase in HDL
			Reduction in LDL and TG
Pathan et al. [142]	2010	30 males	Increase in HDL
			Reduction in TC, LDL, VLDL and TG
Dowod [131]	2004	60	Reduction in LDL
Shahsavan et al. [143]	2015	89	Reduction in TC and HDL
			No change in LDL, TG or LDL/HDL
Khan et al. [144]	2017	Pakistan	Increase in LDL
			Reduction in HDL
Indra et al. [145]	2007	Indonesia	Reduction in TC, LDL and TG

TC: Total cholesterol, TG: Triglycerides, HDL: High density lipoprotein, LDL: Low density lipoprotein, VLDL: Very low density lipoprotein.
